# Data on microbial community composition of sludge from high altitude wastewater treatment plants determined by 16S rRNA gene sequencing

**DOI:** 10.1016/j.dib.2019.103739

**Published:** 2019-03-07

**Authors:** Chen Chen, Xiaochun Peng, Shan Huang, Yuannan Wang, Shumei Liao, Yang Wei

**Affiliations:** a South China Institute of Environmental Sciences, Ministry of Environmental Protection, Guangzhou, 510535, China; b South China Advanced Environmental Technologies Co., LTD, Guangzhou, 51000, China; c Department of Civil and Environmental Engineering, Princeton University, Princeton, NJ 08544, USA; d School of Environment and Safety Engineering, North University of China, Taiyuan, 030051, China

## Abstract

This work presented the sequences of activated sludge from two municipal wastewater treatment plants (WWTPs) located in a high altitude Plateau in Tibet, China (∼3650 m above the sea level). Sequencing data are the 16S rRNA gene amplicons of V4—V5 region that sequenced on an Illumina HiSeq PE250 platform. Data presented here include detail description and water quality parameters of the WWTPs as well as results of 16S rRNA gene sequences from their active sludges. The core microbial communities in the WWTPs were shown at the taxonomic level of phylum, class, order, family, genus and species. The sequencing data have been deposited in NCBI BioProject PRJNA477990 with the Biosample accessions SAMN09488330-SAMN09488338. The annotation of OTU table at the genus level was assessable on Zenodo (https://zenodo.org/record/2105899#.XA0vQPZuJyw).

**Table:** Specifications table

Subject area	*Biology*
More specific subject area	*Bacterial wastewater treatment*
Type of data	*Graph of location; table of chemicals; graphs of 16S rRNA sequences; annotation at the genus level*
How data was acquired	*DNA sequencing using Illumina HiSeq PE250 platform*
Data format	*Raw and filtered*
Experimental factors	*DNA extracted from activated sludge*
Experimental features	*Activated sludge was collected from high altitude wastewater treatment plants in Tibetan Plateau. The 16S rRNA gene amplicon sequencing of V4*—*V5 section were performed using Illumina HiSeq PE250 platform.*
Data source location	*Lasha, China; ∼3650 m above the sea level.*
Data accessibility	*Data about microbial community structure are available with the article. The sequencing data have been deposited in NCBI BioProject PRJNA477990 (BioSample accession number SAMN09488330 to SAMN09488338). The annotation of OTU table at the genus level was assessable on* Zenodo (https://zenodo.org/record/2105899#.XA0vQPZuJyw).

**Table:** 

**Value of the data** • This dataset includes microbial surveys from two wastewater treatment plants (WWTPs) located at a high altitude Plateau in Tibet, China (∼3650 m). • Sequencing data can be used by other researchers to compare the core microbial community structure between WWTPs in high altitude regions and other locations. • The data is publicly available for the comparison of microbial community structures within two WWTPs operated at different processes (cyclic activated sludge system vs anaerobic-anoxic-aerobic process) in high altitude regions. • High altitude region has low oxygen level, which forms a natural low oxygen condition for microbial communities, thus these data can be used for the comparative studies related to wastewater treatment that operated under low dissolved oxygen condition.

## 1 Data

**Fig. 1:**
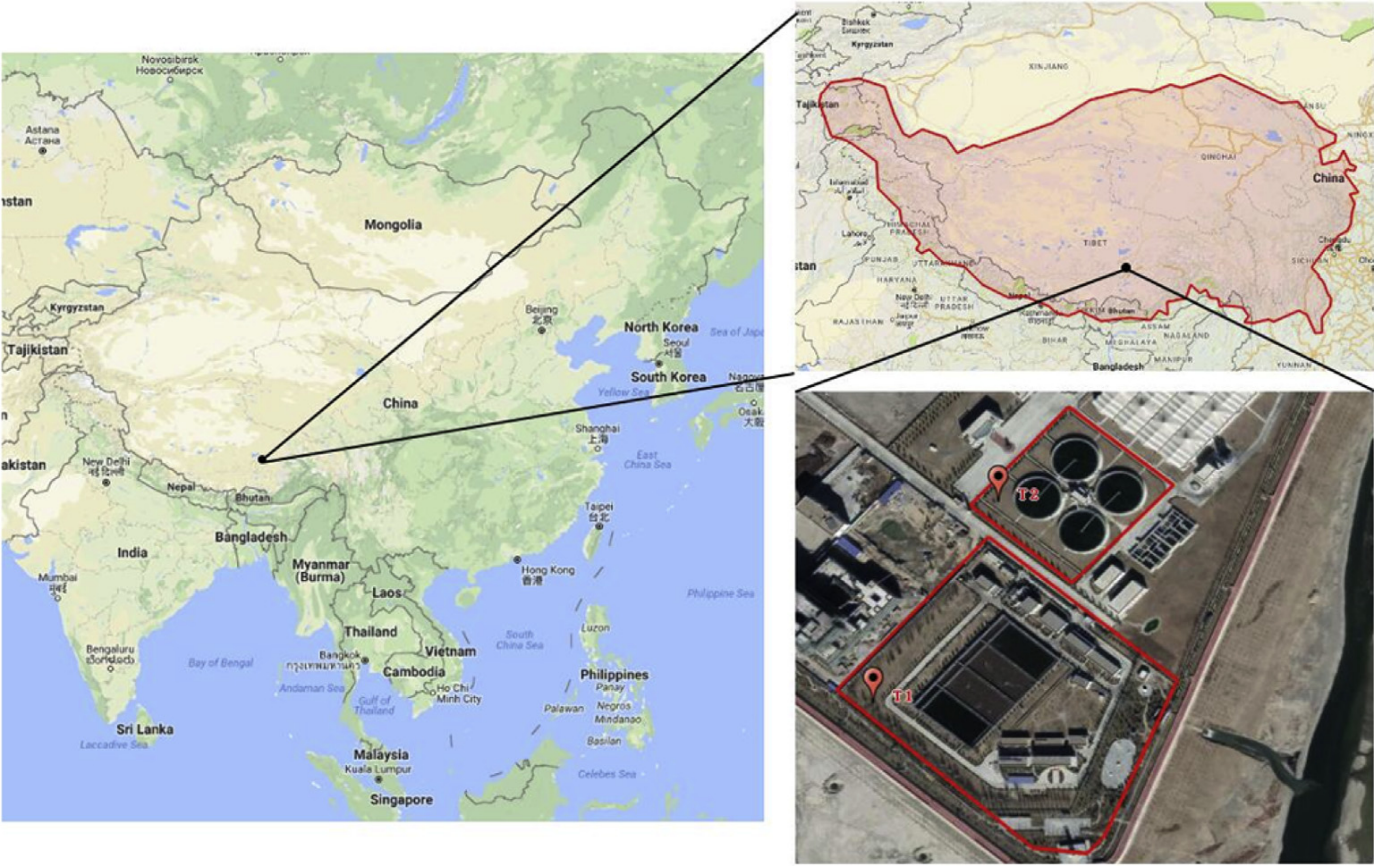
Location of wastewater treatment plants. T1 (91°0′49″E, 29°37′19″N) is applied for cyclic activated sludge system, while T2 (91°0′51″E, 29°37′26″N) is operated at anaerobic-anoxic-aerobic (A2O) process.

**Fig. 2:**
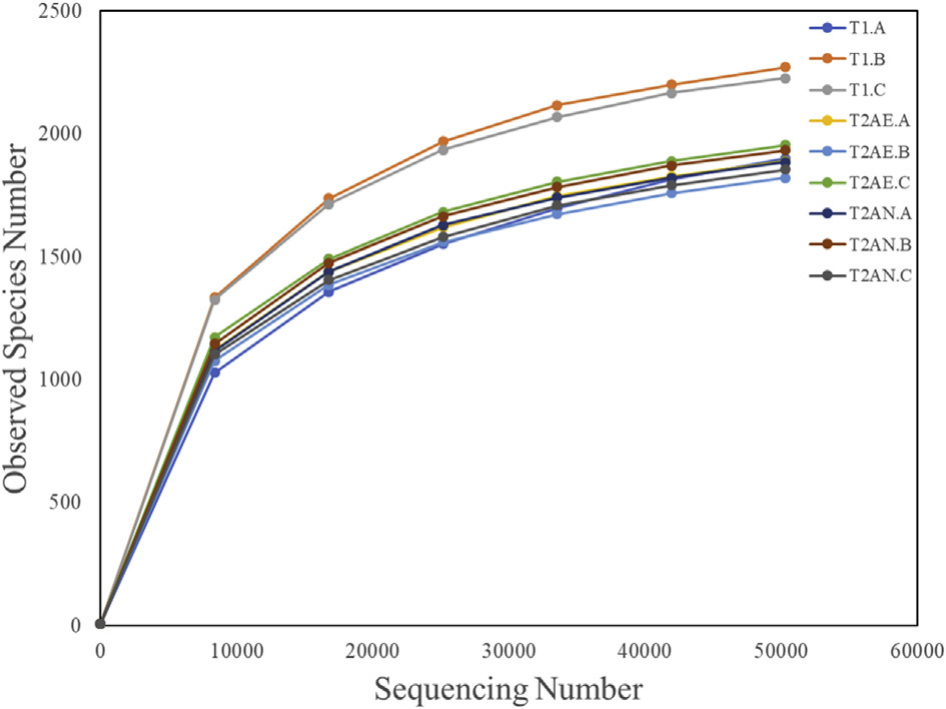
Rarefaction curve analysis in 9 sludge samples, which showed a higher species richness in T1 than T2.

**Fig. 3:**
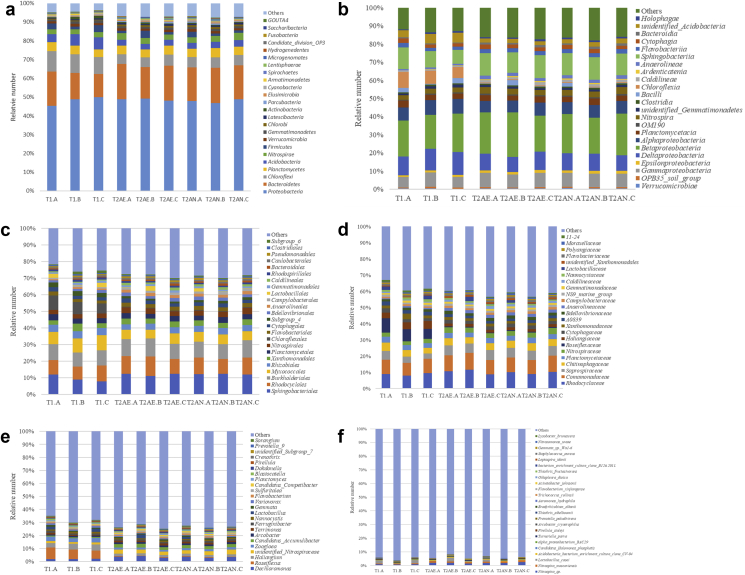
Top 25 microbial communities in wastewater treatment plants at the taxonomic levels (a: phylum; b: class; c: order; d: family; e: genus; f: species).

Data on microbial communities of activated sludge from two municipal wastewater treatment plants (WWTPs) located in a high altitude Plateau in Tibet, China (∼3650 m above the sea level) are presented. T1 is applied for cyclic activated sludge system (CASS), while T2 is operated at anaerobic-anoxic-aerobic (A^2^O) process. Locations of the two WWTPs are shown in Fig. 1. Dataset contains several water quality parameters, which including suspended solids (SS), chemical oxygen demand (COD), total nitrogen (TN), total phosphate (TP), ammonium, and pH, collected from inflow and outflow of each WWTPs. Data present include 16S rRNA gene amplicons of V4—V5 region that sequenced on an Illumina HiSeq PE250 platform, and then annotated using the 16S-Silva database (Fig. 2). Data of normalized OTU relative number of each sample are presented at the taxonomic levels of phylum, class, order, family, genus and species (Fig. 3 a-f). The raw and filtered dataset are deposited in NCBI BioProject PRJNA477990 with the BioSample accession number SAMN09488330 to SAMN09488338. The annotation of OTU table at the genus level was assessable on Zenodo (https://zenodo.org/record/2105899#.XA0vQPZuJyw).

## 2 Experimental design, materials, and methods

### 2.1 Description of WWTPs

The WWTPs located in Tibet Plateau, which is called “the Roof of the World”, because it is the world's highest plateau with an average of 4,500 m above sea level. Two WWTPs are set up beside each other and marked as T1 (91°0′49″E, 29°37′19″N) and T2 (91°0′51″E, 29°37′26″N), respectively. Location of Tibet Plateau and WWTPs are shown in Fig. 1.

These two WWTPs are established to treat the urban wastewater from the city of Lhasa, China (∼3650 m), which has the population of 5.59 × 10^6^. T1 has the flow of 5.32 × 10^5^ m^3^ daily, which fixed in its maximum daily capacity. Wastewater is first filtered through roughing and fine grid to remove particles, then operated with CASS process in the bioreactor, with an intermittent aeration operation as 2 hour aeration in each 4 hour cycle. The parameters of inflow and outflow are determined daily with the recommended methods from Discharge standard of pollutants for municipal wastewater treatment plant [1]. The detection of SS was via gravimetric method [2], COD_Cr_ via dichromate method [3], TN via alkaline potassium persulfate digestion-UV spectrophotometric method [4], ammonium via distillation and titration [5], TP via ammonium molybdate spectrophotometric method [6], and pH via glass electrode method [7]. The average of each parameters was listed in Table 1, and the removal efficiencies of SS, COD_Cr_, TN, NH_4_^+^—N and TP were 95.4%, 90.5%, 89.0%, 93.8%, 56.3%, respectively. T2 has treated 7.76 × 10^5^ m^3^ wastewater daily, about 60% of its maximum daily capacity. Inflow is also filtered through roughing and fine grids as in T1, while T2 utilizes A^2^O process with internal cycle of 100%–200% for the further treatment. The aerobic tank and anaerobic tank were marked as T2AE and T2AN in this study. All parameters of inflow and outflow are determined daily as described above (Table 1). The removal efficiencies of SS, COD_Cr_, TN, NH_4_^+^—N and TP were 97.9%, 94.2%, 89.0%, 91.4% and 63.5%, respectively.

**Table 1:** Characters of wastewater treatment plants T1 and T2.

	Treatment process	maximum daily capacity (m^3^ d^−1^)	Flow (m^3^ d^−1^)	Inflow (mg L^–1^)						Outflow (mg L^–1^)					
				SS	COD_cr_	TN	NH_4_—N	TP	pH	SS	COD_cr_	TN	NH_4_—N	TP	pH
T1	CASS	5.32 × 10^5^	5.32 × 10^5^	144	123	15.5	5.96	1.19	7.02	6.57	11.67	1.70	0.37	0.52	7.08
T2	A^2^O	13.0 × 10^5^	7.76 × 10^5^	102	130	15.5	6.02	0.85	6.86	2.13	7.55	1.70	0.52	0.31	7.12

CASS, cyclic activated sludge system; A^2^O, anaerobic-anoxic-aerobic process; SS: suspended solids; COD: chemical oxygen demand; TN: total nitrogen; TP: total phosphate.

### 2.2 DNA extraction, amplification and sequencing

For each bioreactors (T1, T2AE and T2AN), 3 replicates of 50 mL sludge sample were taken and labelled as A, B, and C, which contributed to a total of 9 sludge samples for DNA sequencing. DNA samples were extracted from all 9 collected sludge samples using FastDNA^®^ spin kit for soil (MP Biomedicals, USA) following the manuscript of the manufacture. The 16S rRNA gene of V4—V5 was amplified using primers 515f-926r [8], [9] following methods suggested by Caporaso et al. [10]. Each 30 μL PCR mixture was composed of 15 μL of Phusion^®^ High-Fidelity PCR Master Mix (New England Biolabs), 3 μL of each primers (6 μM final concentration), 10 μL of gDNA (5–10 ng) and 2 μL ddH_2_O. The PCR program was initiated for 1 min at 98 °C; followed by 30 cycles of 10 s at 98 °C, 30 s at annealing temperatures of 50 °C, and 30 s at 72 °C; then a final extension of 5 min at 72 °C. All PCR products were quantified and purified before sequencing. Then, sequencing libraries were generated using TruSeq^®^ DNA PCR-Free Sample Preparation Kit (Illumina, USA) following manufacturer's recommendations. The library quality was assessed on the Qubit@ 2.0 Fluorometer (Thermo Scientific, USA) and Agilent Bioanalyzer 2100 system (Agilent Technologies, USA). At last, the library was sequenced on an Illumina PE250 platform and 250–500 bp paired-end reads were generated. All amplicon sequencings were conducted on an Illumina PE250 platform at Novogene Co., Beijing, China. A total of 81,409–99,124 raw sequences was obtained for each sample. The raw and filtered sequencing data have been deposited in NCBI BioProject PRJNA477990, and the SRA accesion number of each sample was listed in Table 2.

**Table 2:** Sample identification and SRA accession number.

Sample identification	Accession Number
T1.A	SAMN09488330
T1.B	SAMN09488331
T1.C	SAMN09488332
T2.A	SAMN09488333
T2.B	SAMN09488334
T2.C	SAMN09488335
T3.A	SAMN09488336
T3.B	SAMN09488337
T3.C	SAMN09488338

### 2.3 Paired-end reads assembly and quality control

Paired-end reads were first assigned to samples, then split and assembled by FLASH (V1.2.7) (overlap minimum of 10 bp, maximum mismatch density of 0.25) [11]. To get high qualified reads, raw tags were filtered by QIIME(V1.7.0) [10], then compared to Gold database [12] as reference database to detect and remove chimeric sequences by UCHIME algorithm (Drive 5) [13]. A total of 71,584–85,487 qualified reads was created for the next annotation step.

### 2.4 Annotation and OTU abundance

Operational taxonomic units (OTUs) were clustered with a 97% similarity cut-off using the Uparse software (Uparse v7.0.100, http://drive5.com/uparse/) [13]. After dereplication, abundance sort, discarding singleton, clustering, the OTU table was created with 1900–2482 OTUs for each sample.

A representative sequence for each OTU was screened for further annotation. The taxonomy of each 16S rRNA gene sequence was analyzed with Muther (version v.1.30.1) against the 16S-Silva SSUrRNA database using a confidence threshold of 0.8–1, which provides the taxonomic information from kingdom level to species level [14], [15]. OTUs abundance information was normalized using a standard of sequence number corresponding to the sample with the least sequences. In this case, the relative number of the microbial community was performed based on the normalized data, which was present at the taxonomic level (phylum to species).
