# Supplementary figures and images for: Data on microbial community composition of sludge from high altitude wastewater treatment plants determined by 16S rRNA gene sequencing
Source: Data Brief. 2019 Mar 7;23:103739. doi: 10.1016/j.dib.2019.103739 (PMC6660466; doi:10.1016/j.dib.2019.103739)

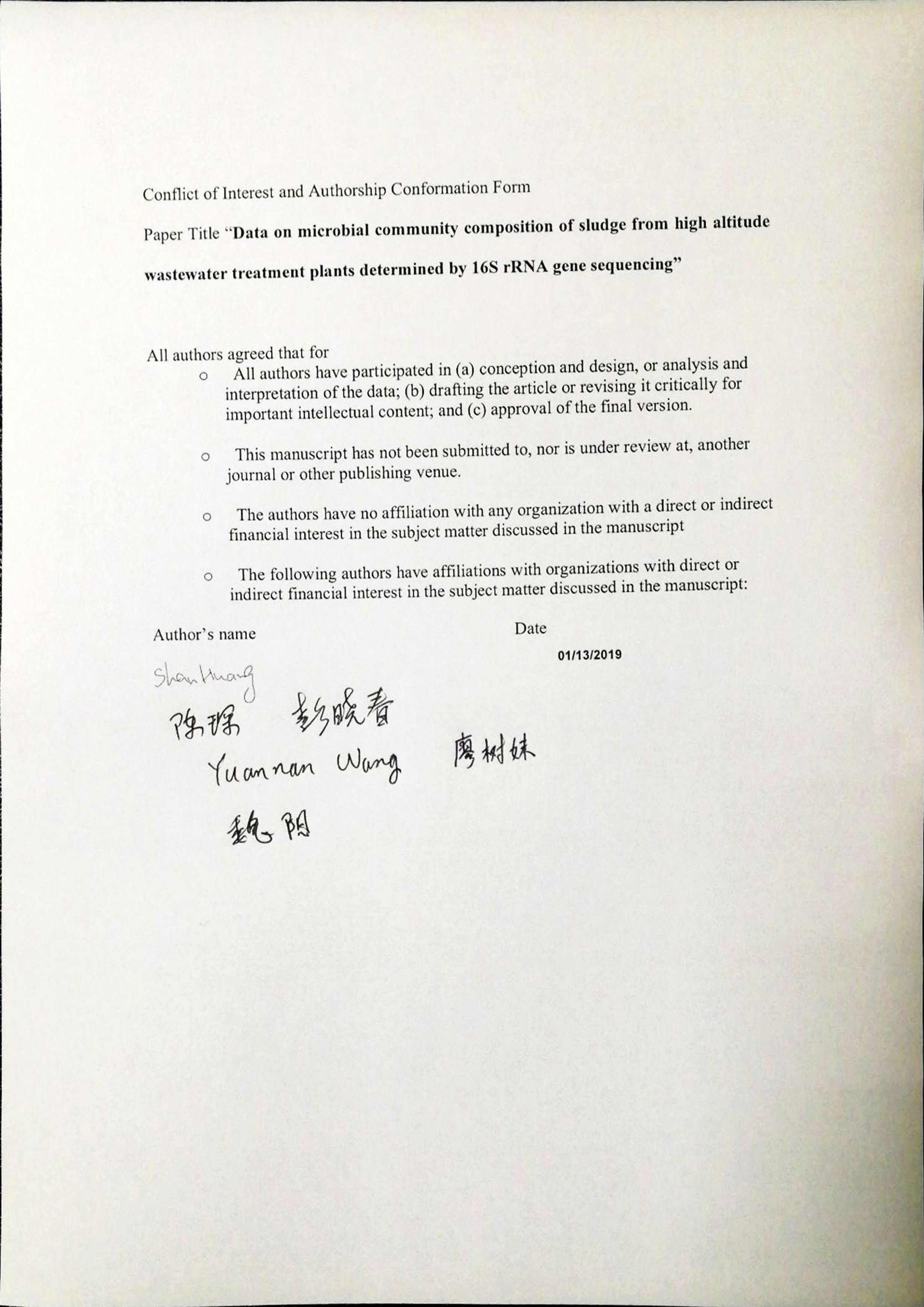

Supplement: Supplementary file 1 — Multimedia component 1 [file figs1.jpg]
